# Folic acid inhibits COLO-205 colon cancer cell proliferation through activating the FRα/c-SRC/ERK1/2/NFκB/TP53 pathway: *in vitro* and *in vivo* studies

**DOI:** 10.1038/srep11187

**Published:** 2015-06-09

**Authors:** Chun-Ting Kuo, Chieh Chang, Wen-Sen Lee

**Affiliations:** 1 Graduate Institute of Medical Sciences, College of Medicine, Taipei Medical University, Taipei 110, Taiwan; 2 School of Pharmacy, Taipei Medical University, Taipei 110, Taiwan; 3 Department of Physiology, School of Medicine, College of Medicine, Taipei Medical University, Taipei 110, Taiwan; 4 Cancer Research Center, Taipei Medical University Hospital, Taipei 110, Taiwan

## Abstract

To investigate the molecular mechanism underlying folic acid (FA)-induced anti-colon caner activity, we showed that FA caused G0/G1 arrest in COLO-205. FA activated the proto-oncogene tyrosine-protein kinase Src (c-SRC)-mediated signaling pathway to enhance nuclear factor of kappa light polypeptide gene enhancer in B-cells (NFκB) nuclear translocation and binding onto the *tumor protein p53* (*TP53*) gene promoter, and up-regulated expressions of TP53, cyclin-dependent kinase inhibitor 1A (CDKN1A) and cyclin-dependent kinase inhibitor 1B (CDKN1B). Knock-down of TP53 abolished FA-induced increases in the levels of CDKN1A and CDKN1B protein and G0/G1 arrest in COLO-205. Knock-down of folate receptor alpha (FRα) abolished FA-induced activations in the c-SRC-mediated pathway and increases in the levels of CDKN1A, CDKN1B and TP53 protein. These data suggest that FA inhibited COLO-205 proliferation through activating the FRα/c-SRC/mitogen-activated protein kinase 3/1 (ERK1/2)/NFκB/TP53 pathway-mediated up-regulations of CDKN1A and CDKN1B protein. *In vivo* studies demonstrated that daily i.p. injections of FA led to profound regression of the COLO-205 tumors and prolong the lifespan. In these tumors, the levels of CDKN1A, CDKN1B and TP53 protein were increased and von willebrand factor (VWF) protein levels were decreased. These findings suggest that FA inhibits COLO-205 colon cancer growth through anti-cancer cell proliferation and anti-angiogenesis.

Folate, a water-soluble vitamin B9, is an important factor for DNA synthesis, cell proliferation, and a number of metabolic pathways^1^,^2^. Most mammals are unable to synthesize folate; therefore, the folate requirement must be obtained from dietary or supplementary sources. FA used as nutritional supplement for folate is the fully oxidized monoglutamyl form of folate^3^. A deficiency of folate in tissues results in ineffective DNA synthesis and reducing cell proliferation^4^, and has been implicated in various diseases, including atherosclerosis^5^, anemia, neural tube defects^6^,^7^ and cancer^8^,^9^,^10^,^11^.

Folate has been demonstrated to exert an inverse relationship between the risks of some malignancies including cancer of colon, stomach, pancreas, lung, ovary, breast and leukemia^12^,^13^,^14^. Epidemiological and clinical studies also showed that dietary folate supplement might decrease the risk of colorectal cancer and be involved in DNA methylation of *TP53*^13^, suggesting that folate might exert protective effects on colorectal cancer^15^,^16^. However, its underlying molecular mechanism is still not well known.

Previously, we demonstrated that FA inhibits human endothelial cell proliferation though activating the c-SRC/ERK 2/NFκB/TP53 pathway mediated by FRγ^17^, suggesting a novel role of FRγ for anti-angiogenesis. In this investigation, we identified that FA bound to FRα, subsequently increasing the levels of CDKN1A and CDKN1B protein through activating the c-SRC/ERK1/2/NFκB/TP53 pathway, and eventually inhibited the proliferation of COLO-205.

## Results

### Effects of FA on cell proliferation and the levels of cell cycle regulatory proteins in COLO-205

**Figure 1:**
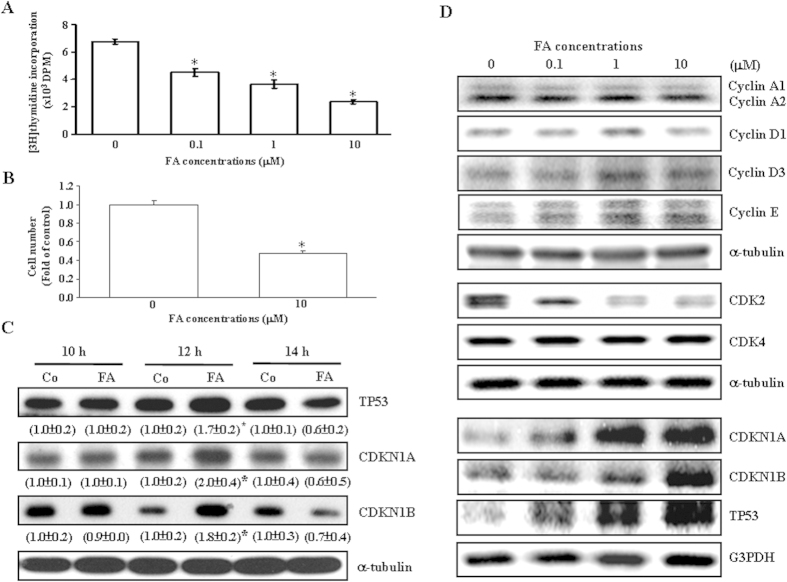
Effects of FA on cell proliferation and the levels of cell cycle regulatory proteins in COLO-205. (**A**) Concentration-dependent inhibition of [3H]thymidine incorporation into COLO-205 by FA. Cells were incubated with FA (0.1–10 μM) for 21 h and [3H]thymidine was added only for the last 3 h of this incubation. Values represent the means ± s.e.mean. (n = 4). * *p* < 0.05 different from control. (**B**) FA at a concentration of 10 μM significantly reduced the cell number in COLO-205. Media with or without FA were changed daily until cell counting. Cells were allowed to grow for 6 days after vehicle (control) or FA treatment. Values represent the means ± s.e.mean. (n = 4). * *p* < 0.05 different from control. (**C**) FA up-regulates the expression of TP53, CDKN1A and CDKN1B in COLO-205 in a time-dependent manner. Protein was extracted from the cultured COLO-205 at indicated time points after FA treatment and probed with proper dilutions of specific antibodies. Values (means ± s.e.mean.) shown in parentheses represent the relative protein abundance of CDKN1A, CDKN1B and TP53, which has been normalized with corresponding α–tubulin and expressed as fold of its own control. Three samples were analyzed in each group. * *p* < 0.05 different from its own control. (**D**) FA (0.1−10 μM) concentration-dependently decreased the levels of CDK2 protein, increased the levels of CDKN1A, CDKN1B and TP53 protein, but not significantly affected the levels of cyclin A, D1, D3, and E, and CDK4 protein. Protein was extracted from the cultured COLO-205 at 12 h after FA treatment and probed with proper dilutions of specific antibodies. The gels have been run in the same experimental conditions and the cropped blots were shown. The entire gel pictures of Fig. 1C were shown in the Supplemental Figure 1A.

Initially, we examined the effect of FA on the growth of COLO-205 by examining changes in [3H]thymidine incorporation. As shown in Fig. 1A, FA (0.1–10 μM) concentration-dependently inhibited [3H]thymidine incorporation into COLO-205. We further examined the effect of FA on the cell number of COLO-205. Daily treatment with FA (10 μM) for 6 days significantly decreased the cell number in COLO-205 (Fig. 1B). FA-induced decreases in the cell growth rate were consistent with the inhibitory effect of FA on [3H]thymidine incorporation. The FA-induced reduction in [3H]thymidine incorporation in COLO-205 can be due to retardation of cell cycle or cell death. To confirm that the results of our studies of DNA synthesis and cellular proliferation in COLO-205 were not due to cell death caused by FA treatment, we conducted Trypan blue assays to examine the cell viability by treating the cell with FA for 6 days at a concentration of 10 μM used in the studies of [3H]thymidine incorporation. The results showed that FA did not cause any significant cell death as compared with the cell treated with vehicle (0.02 ± 0.01% for FA-treated group vs. 0.03 ± 0.01% for vehicle-treated group). Since FA arrested the cell cycle of COLO-205 at the G0/G1 phase, we examined the effect of FA on the levels of G1 phase regulatory proteins in COLO-205. FA-induced increases in the levels of TP53, CDKN1A, and CDKN1B were observed at 12 h after FA treatment (Fig. 1C). As shown in Fig. 1D, FA (0.1–10 μM) concentration-dependently decreased the levels of CDK2 protein, increased the levels of CDKN1A, CDKN1B and TP53 protein, but not significantly affected the levels of cyclin A, D1, D3, and E, and CDK4 protein.

### Up-regulation of TP53 contributes to FA-induced CDKN1A and CDKN1B up-regulations and G0/G1 arrest in COLO-205

**Figure 2:**
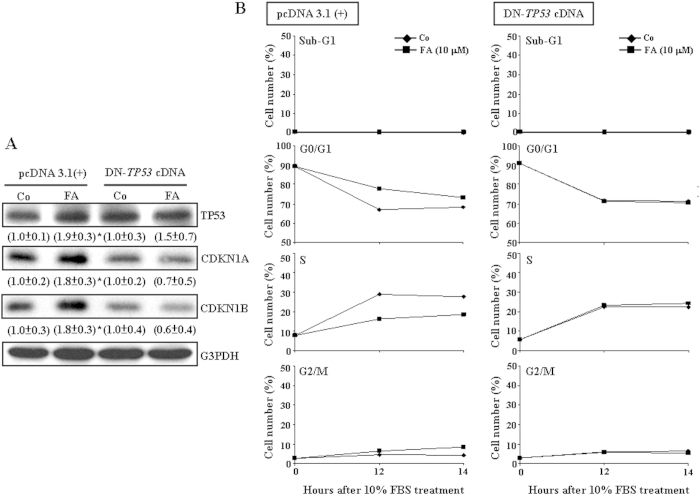
FA induces TP53-dependent up-regulations in CDKN1A and CDKN1B and the G0/G1 arrest in COLO-205. (**A**) Pre-transfection with DN-*TP53* cDNA prevented FA-induced increases in the levels of CDKN1A and CDKN1B protein. In the control group, the cell was transfected with pcDNA 3.1(+) expression vector. The gels have been run in the same experimental conditions and the cropped blots were shown. The entire gel pictures of Fig. 2A were shown in the Supplemental Fig. 1B. Values (means ± s.e.mean.) shown in parentheses represent the relative protein abundance of CDKN1A, CDKN1B and TP53, which has been normalized with corresponding G3PDH and expressed as fold of its own control. Three samples were analyzed in each group * *p* < 0.05 different from it own control. (**B**) Pre-transfection with DN-*TP53* cDNA prevented the FA-induced G0/G1 arrest in COLO-205. Data are representative of 2 independent experiments with similar results. Co, control; DN-*TP53* cDNA, dominant negative *TP53* cDNA.

To further investigate whether FA-induced increases in the levels of CDKN1A and CDKN1B protein and the G0/G1 arrest were regulated by TP53, the dominant negative *TP53* cDNA (DN-*TP53* cDNA), which is the mutant construct for DNA binding site of *TP53*^18^, was transfected into COLO-205 followed by FA treatment. As illustrated in Fig. 2A, pre-transfection with DN-*TP53* cDNA abolished FA-induced increases in the levels of TP53, CDKN1A and CDKN1B protein. Moreover, pre-transfection with DN-*TP53* cDNA also abolished the FA-induced G0/G1 arrest in COLO-205 (Fig. 2B).

### Signaling pathway involved in the FA-induced up-regulation of TP53 in COLO-205

**Figure 3:**
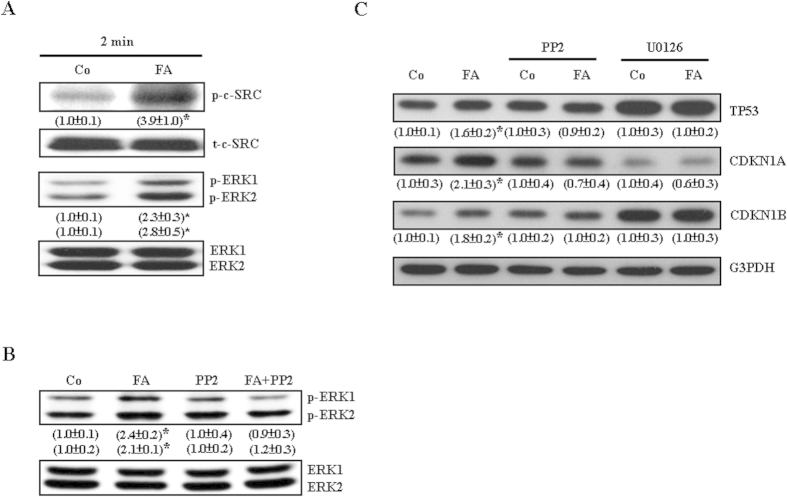
FA-induced activations in c-SRC and ERK1/2 contribute to FA-induced up-regulations in cell cycle inhibitory proteins in COLO-205. (**A**) Treatment of COLO-205 with FA (10 μM) for 2 min significantly increased the levels of p-c-SRC and p-ERK1/2 protein. (**B**) Pre-treatment with the c-SRC inhibitor, PP2 (200 nM), prevented FA-induced increases in the levels of p-ERK1/2 protein. (**C**) Pre-treatment of COLO-205 with PP2 (200 nM) or the ERK1/2 inhibitor, U0126 (5 μM), prevented FA-induced up-regulations in TP53, CDKN1A and CDKN1B protein. The cell was pre-treated with PP2 or U0126 for 1 h followed by FA (10 μM) treatment for an additional 12 h, and then processed for protein extraction and Western blot analyses. The gels have been run in the same experimental conditions and the cropped blots were shown. The entire gel pictures of Fig. 3A–C were shown in the Supplemental Fig. 2A–C. Values (means ± s.e.mean.) shown in parentheses represent the relative protein abundance, which has been normalized with corresponding total protein or G3PDH and expressed as fold of its own control. Three samples were analyzed in each group. * *p* < 0.05 different from it own control. Co, Control.

**Figure 4:**
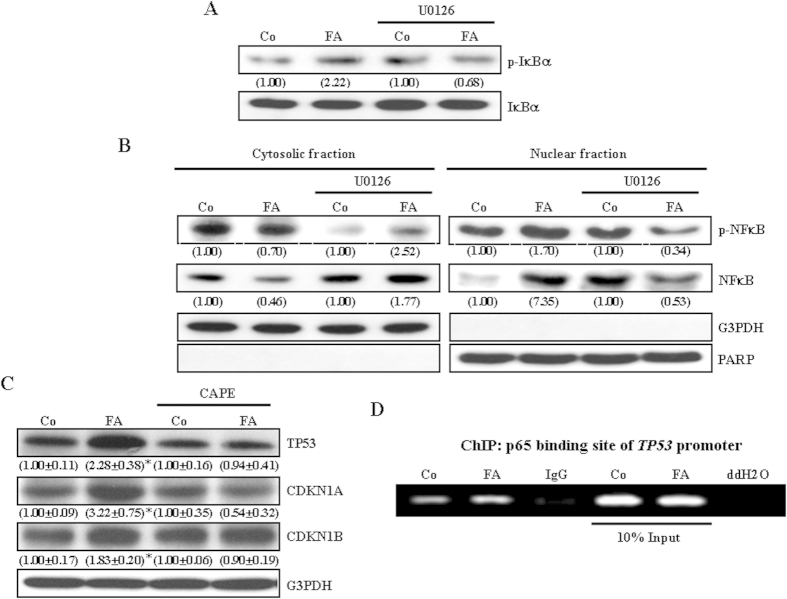
Activation of NFκB contributes to FA-induced up-regulations in cell cycle inhibitory proteins in COLO-205. Pre-treatment with U0126 (5 μM) prevented FA-induced increases in IκBα phosphorylation (**A**), and nuclear translocation of NFκB and p-NFκB. (**B**). Values (means ± s.e.mean.) shown in parentheses represent the relative protein abundance, which has been normalized with corresponding total protein in (**A**), PARP (for nuclear fraction) or G3PDH (for cytosolic fraction) in (**B**) and expressed as fold of its own control. (**C**) Pre-treatment of COLO-205 with the NFκB inhibitor, CAPE (5 μM), prevented FA-induced increases in the levels of TP53, CDKN1A and CDKN1B protein. The cell was treated with CAPE for 1 h followed by FA (10 μM) treatment for an additional 12 h, and then processed for protein extraction and Western blot analyses. The gels have been run in the same experimental conditions and the cropped blots were shown. The entire gel pictures of Fig. 4A–C were shown in the Supplemental Fig. 3A–C. Three samples were analyzed in each group, and values represent the means ± s.e.mean. * *p* < 0.05 different from its own control. (**D**) FA (10 μM) treatment increased the binding of p65 onto the *TP53* promoter. CAPE, Caffeic acid phenethylester; ChIP, chromatin immunoprecipiation; Co, Control; ddH2O, double-distilled water.

We further investigated the signaling pathway involved in the FA-induced TP53 up-regulation in COLO-205. Since activation of the c-SRC/ERK 2/NFκB-mediated pathway has been demonstrated to be involved in the FA-induced TP53 up-regulation and proliferation inhibition in HUVEC^17^, we examined whether activations in c-SRC and ERK are also involved in FA-induced up-regulations in the levels of TP53, CDKN1A, and CDKN1B protein in COLO-205. Treatment with FA (10 μM) for 2 min increased in the levels of p-c-SRC and p-ERK1/2 protein in COLO-205 (Fig. 3A), suggesting the involvement of c-SRC and ERK1/2 activation in FA-induced up-regulations in TP53, CDKN1A, and CDKN1B protein in COLO-205. Pre-treatment of COLO-205 with the c-SRC inhibitor, 4-amino-5-(4-chlorophenyl)-7-(t-butyl) pyrazolo[3,4-d]pyramidine (PP2, 200 nM), prevented FA-induced increases in the level of ERK1/2 protein (Fig. 3B), suggesting that c-SRC is the upstream molecule of ERK1/2. Moreover, pre-treatment with PP2 or the ERK inhibitor (U0126, 5 μM) prevented FA-induced increases in the levels of TP53, CDKN1A, and CDKN1B protein in COLO-205 (Fig. 3C). Pre-treatment with U0126 also prevented FA-induced increases in the level of phosphoryated NF-kappa-B inhibitor alpha (p-IκBα) protein (Fig. 4A) and nuclear translocation of NFκB and p-NFκB (Fig. 4B). We also examined whether nuclear translocation of NFκB, which has been implicated in regulating the expression of TP53 in HUVEC^19^, is involved in the FA-induced up-regulation of TP53. As shown in Fig. 4C, pre-treatment with the NFκB inhibitor, CAPE (5 μM), prevented FA-induced increases in the levels of TP53, CDKN1A and CDKN1B protein in COLO-205. Moreover, FA treatment increased the binding of NFκB (p65) onto the *TP53* promoter (Fig. 4D). These data suggest that activation of the c-SRC/ERK1/2/NFκB-mediated pathway was involved in FA-induced increases in the levels of TP53, CDKN1A and CDKN1B protein.

### Involvement of the FRα isoform in the FA-induced activations of c-SRC and increases in the levels of cell cycle inhibitory proteins in COLO-205

**Figure 5:**
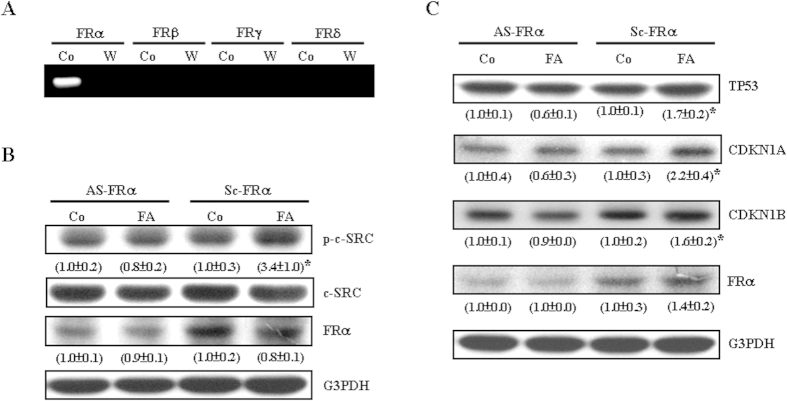
Involvement of the FRα isoform in the FA-induced activation of c-SRC and increases in cell cycle inhibitory proteins in COLO-205. (**A**) RT-PCR demonstrated that only the FRα isoform was detected in COLO-205. Knock-down of FRα with antisense oligonucleotide (10 μM) abolished the FA-induced c-SRC activation (**B**) and FA-induced increases in TP53, CDKN1A, and CDKN1B protein (**C**) in COLO-205. The gels have been run in the same experimental conditions and the cropped blots were shown. The entire gel pictures of Fig. 5B–C were shown in the Supplemental Figure 4A–B. Values (means ± s.e.mean.) shown in parentheses represent the relative protein abundance, which has been normalized with corresponding total protein or G3PDH and expressed as fold of its own control. Three samples were analyzed in each group. * *p* < 0.05 different from its own control. AS, antisense oligonucleotide; Co, control; Sc, scramble oligonucleotide; W, water.

Previously, we demonstrated that the FA-induced proliferation inhibition in HUVEC is mediated through activating the FRγ isoform^17^. In the present study, we examined which isoforms of FR were expressed in COLO-205. As shown in Fig. 5A, only the FRα isoform was detected in COLO-205. We next examined whether activation of FRα is involved in the FA-induced c-SRC activation. As illustrated in Fig. 5B, knock-down of FRα abolished the FA-induced activation in c-SRC, suggesting that FRα is involved in the FA-induced c-SRC activation. We also examined whether knock-down of FRα could abolish FA-induced increases in the levels of TP53, CDKN1A and CDKN1B protein. As shown in Fig. 5C, knock-down of FRα abolished FA-induced increases in the levels of TP53, CDKN1A, and CDKN1B protein.

### FA causes tumor regression and prolongs the life span

**Figure 6:**
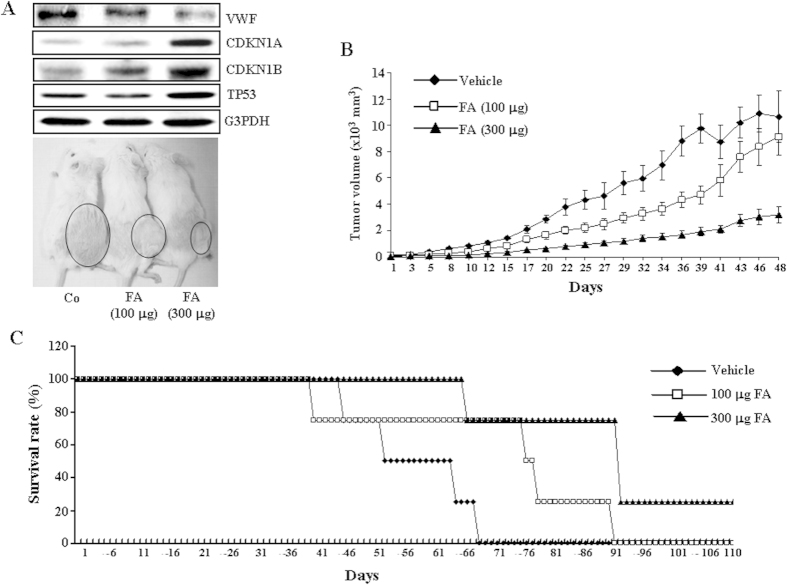
Effect of FA on tumor growth and survival rate in tumor bearing mice. (**A**) FA dose-dependently reduces the growth rate of tumors in SCID mice. Gross appearance of subcutaneous tumors after treatment with FA or vehicle for 48 days. The upper panels show that FA treatment causes a decrease in the level of VWF protein and increases in the levels of CDKN1A, CDKN1B and TP53 protein. The gels have been run in the same experimental conditions and the cropped blots were shown. The entire gel pictures of Fig. 6A were shown in the Supplemental Figure 4C. (**B**) Average tumor volume of vehicle-treated (diamond) vs. FA-treated (square, 100 μg FA; triangle, 300 μg FA). FA dose-dependently reduces the tumor volume. (**C**) FA dose-dependently prolongs the lifespan of mice bearing the COLO-205 tumor. Co, control; VWF, Von Willebrand factor.

Given the inhibition by FA of the growth of transformed colon cells in culture, we next determined whether administration of FA could affect the growth of tumors derived from human colon cancer cells in an *in vivo* setting. As illustrated in Fig. 6A,B, intraperitoneal injection of FA (0–300 μg) dose-dependently reduced the tumor volume. Moreover, the lifespan of FA-injected tumor bearing mice was lengthened as compared with the control mice injected with vehicle (Fig. 6C). The degree of prolongation in lifespan was proportional to the dose of FA injected. Following upon the observation of cell cycle arrest and inhibition of angiogenesis, we examined the FA effect on the cell cycle and angiogenesis of the solid tumor derived from the implanted COLO-205. The levels of VWF protein, a marker of endothelial cells, were decreased and CDKN1A, CDKN1B and TP53 protein were increased in the tumor isolated from the FA-treated mouse (Fig. 6A, top panel). These findings suggest that the inhibition of cell cycle progression in COLO-205 and impairment of the angiogenesis process eventuated in the regression of the FA-treated tumors.

### Effects of FA on the growth of HT-29 and LoVo colon cancer cell lines

**Figure 7:**
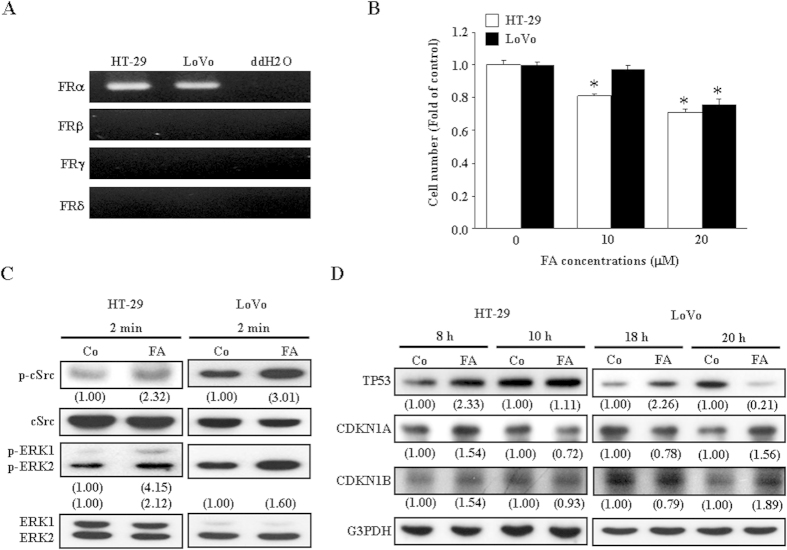
Effects of FA on the growth of HT-29 and LoVo colon cancer cell lines. (**A**) RT-PCR demonstrated that only the FRα isoform was detected in both HT-29 and LoVo. (**B**) Treatment with FA reduced the growth of both HT-29 and LoVo. Media with or without FA were changed daily until cell counting. Cells were allowed to grow for 4 days after vehicle (control) or FA treatment. Values represent the means ± s.e.mean. (n = 3). * *p *< 0.05 different from control. (**C**) Treatment with FA for 2 min also increased the levels of p-c-SRC and p-ERK1/2 in HT-29. However, FA only increased the levels of p-c-SRC and p-ERK2, but not p-ERK1, in LoVo. (**D**) Treatment with FA for 8 h increased the levels of CDKN1A, CDKN1B and TP53 protein in HT-29 (left panel). In the FA-treated LoVo, the increased levels of TP53 protein were observed at 18 h after treatment, whereas the increased levels of CDKN1A and CDKN1B were not observed until 20 h after treatment. Values shown in parentheses represent the relative protein abundance, which has been normalized with corresponding total protein or G3PDH and expressed as fold of its own control. The entire gel pictures of Fig. 7C–D were shown in the Supplemental Figure 5A–B. Co, control; ddH2O, double-distilled water.

To examine whether the FA-induced anti-growth effect was also observed in other colon cancer cell lines, the effect of FA on the growth of HT-29 (TP53 mutant) and LoVo (TP53 wild type) were studied. Initially, we examined which FR isoforms were expressed in HT-29 and LoVo. The RT-PCR analysis demonstrated that only the FRα isoform was detected in both HT-29 and LoVo (Fig. 7A). As shown in Fig. 7B, daily treatment with FA for 4 days reduced the growth of both HT-29 and LoVo. We also examined whether the key molecules involved in the FA-induced growth inhibition in COLO-205 are also affected by FA treatment in HT-29 and LoVo. As shown in Fig. 7C, treatment with FA for 2 min also increased the levels of p-c-SRC and p-ERK1/2 in HT-29. However, FA only increased the levels of p-c-SRC and p-ERK2, but not p-ERK1, in LoVo. The effects of FA on the levels of CDKN1A, CDKN1B and TP53 protein were also examined. Since the S phase of the cell cycle of HT-29 and LoVo began at 8 h and 18 h after challenged with serum, respectively^20^,^21^, we examined the changes of the levels of CDKN1A, CDKN1B and TP53 protein at 8 and 10 h (for HT-29) as well as 18 and 20 h (for LoVo) after FA treatment. As illustrated in Fig. 7D, FA increased the levels of CDKN1A, CDKN1B and TP53 protein in HT-29 at 8 h after treatment. In the FA-treated LoVo, the increased levels of TP53 protein were observed at 18 h after treatment, whereas the increased levels of CDKN1A and CDKN1B were not observed until 20 h after treatment.

## Discussion

Previously, we uncovered a completely novel role of FA for anti-angiogenesis and demonstrated that FA inhibits proliferation of vascular endothelial cells through activating the c-SRC/ERK 2/NFκB/TP53 signaling pathway mediated by FRγ^17^. In the present study, we examined the anticancer activity of FA in colon cancer cell lines, COLO-205, HT-29, and LoVo. Our results indicated that treatment with FA reduced the growth of these colon cancer cell lines. Using the COLO-205 as the cell model for further studies, our data suggest that FA inhibited proliferation of COLO-205 through activating the c-SRC/ERK1/2/NFκB/TP53 signaling pathway mediated by FRα and FA nutritional supplementation inhibited COLO-205 colon tumor growth and prolonged the life span of tumor bearing mice.

The FA-induced growth inhibition in COLO-205 is reversible when the cells are transferred to FA free culture media (data not shown). This finding is of considerable relevance because it is generally believed that FA is essential for the synthesis of adenine and thymidine, two of the four nucleic acids that make up genes, DNA, and chromosomes. Previously, we demonstrated that the FA-induced inhibition of DNA synthesis in HUVEC is through increasing CDKN1A and CDKN1B expression, which in turn inhibits the CDK2 activity, and finally impairs the transition from the G1 to the S phase^17^. In the present study, we showed that FA increased the levels of TP53, CDKN1A and CDKN1B protein (Fig. 1C,D). Knock-down of TP53 expression prevented FA-induced increases in the levels of CDKN1A and CDKN1B protein (Fig. 2A) and the G0/G1 arrest (Fig. 2B) in COLO-205, suggesting that FA inhibited COLO-205 proliferation through a TP53-dependent pathway.

While CDKN1A is a known direct target of TP53, the regulation of CDKN1B expression by TP53 is not well established. Previously, we demonstrated that progesterone induces up-regulations in CDKN1B in HUVEC through a TP53-dependent pathway^22^. This conclusion was based on the evidences including that (1) transfection of HUVEC with dominant negative TP53 prevents progesterone-induced increases in the CDKN1B promoter activity and the level of CDKN1B protein; (2) treatment of HUVEC with TP53 siRNA prevents progesterone-induced increases in the level of CDKN1B protein; and (3) progesterone increases the TP53 DNA binding activity on the CDKN1B promoter in HUVEC. A similar phenomenon was also found in the FA-treated HUVEC^17^. The exact molecular mechanism underlying TP53-induced up-regulations in CDKN1B still deserves further investigation.

In the present study, we demonstrated a very narrow window of time for increases in the levels of TP53, CDKN1A and CDKN1B protein. Actually, a similar finding was also observed in the progesterone-induced up-regulations in TP53, CDKN1A and CDKN1B protein in HUVEC^22^. For such a narrow window of time for increases in the levels of TP53, CDKN1A and CDKN1B protein, one possible explanation is a sudden increase in the rate of its degradation. It has been indicated that the half-life of TP53 is primarily regulated by mouse double minute (MDM2), an important negative regulator of TP53. MDM2 targets to ubiquitin residues of TP53 and causes TP53 ubiquitination and proteasomal degradation. MDM2 is one of the TP53’s target genes and any increase of TP53 usually leads to an increase in Mdm2 levels^23^,^24^. We previously demonstrated that progesterone increases the level of MDM2 protein in HUVEC via an up-regulation of TP53^19^. The FA-induced increases in MDM2 were also observed in the FA-treated COLO-205 (Suppl. Fig. 6A). In fact, rapid reductions of the intracellular CDKN1B protein to an undetectable level within 60 min during the progression from the G1 to the S phase through ubiquitin-mediated degradation and proteolytic processing was demonstrated^25^. However, when the cell had a longer exposure to FA treatment the levels of TP53, CDKN1A and CDKN1B protein will be increased again after the MDM2 is degraded. To test this hypothesis, we examined the levels of TP53, CDKN1A and CDKN1B protein in COLO-205 treated with FA for longer periods of time. Indeed, the increased levels of TP53, CDKN1A and CDKN1B protein were observed in the COLO-205 after four days continuous treatment with FA (Suppl. Fig. 6B).

Four FR isoforms including FRα, FRβ, FRγ, and FRδ have been identified. The FRα and FRβ are attached to the membrane by a glycosylphosphatidylinositol (GPI) anchor^26^,^27^. FRα is used as a highly selective tumor marker and may be targeted for the delivery of therapeutic compounds to tumor cells by coupling to derivatives of FA^28^. FRβ is expressed in malignant tissues of epithelial and non-epithelial origin, but not in established cell lines of the same origin^29^. FRγ is expressed in tissues of hematopoietic origin, such as spleen, thymus and bone marrow^30^. The expression pattern of FRδ suggests that it is highly restricted both spatially and temporally^31^. Previously, we identified the expression of FRγ in HUVEC, and demonstrated that FA inhibits HUVEC proliferation through activating the FRγ. In the present study, only FRα mRNA was detected in COLO-205 (Fig. 5A). Knock-down of FRα by antisense oligonucleotide abolished the FA-induced activation of c-SRC (Fig. 5B) and increases in TP53, CDKN1A and CDKN1B protein (Fig. 5C). Moreover, blockade of the activity of c-SRC, ERK1/2, or NFκB prevented FA-induced increases in TP53, CDKN1A and CDKN1B protein. Taken together, these data suggest that FA inhibited proliferation of COLO-205 through activating the FRα/c-SRC/ERK1/2/NFκB/TP53-mediated pathway.

Two theories might explain the potential anti-tumor effects of FA. First, FA might directly inhibit the proliferation of colon cancer cells. The results of our *in vitro* studies do give support for this notion. FA (0.1–10 μM) concentration-dependently inhibited DNA synthesis and decreased the number of COLO-205 cells (Fig. 1A,B). Second, FA might inhibit tumor angiogenesis and the associated tumor growth. Our previous studies demonstrated that FA inhibits the proliferation of cultured vascular endothelial cells *in vitro* and reduces the angiogenesis *in vivo*^17^. In the present study, we found that a lower level of VWF protein, a marker of vascular endothelial cells, was observed in the tumor isolated from the FA-treated mice as compared with those isolated from the vehicle-treated mice (Fig. 6A), suggesting that tumor angiogenesis was reduced in FA-treated mice. To our knowledge, this is the first demonstration that FA inhibits COLO-205 colon cancer growth through inhibiting cancer cell proliferation and angiogenesis.

In the present study, we demonstrate that FA inhibits the progress of COLO-205 both *in vitro* and *in vivo*. The dosage used for *in vivo* study of FA-induced anti-tumor growth study is 100 to 300 μg (i.p. daily), equivalent to 0.3 to 1 mg/kg/day, and was not cytotoxic for the vital organs. Epidemiologic studies have demonstrated that periconceptional dietary supplement of 0.4 to 5 mg FA per day significantly reduces occurrence and recurrence risks for infant neural tube defects without apparent side effects^32^.

In conclusion, the results from the present and our previous studies suggest that FA inhibits COLO-205 colon cancer growth through anti-cancer cell proliferation and anti-angiogenesis. FA induced cell cycle arrest in COLO-205 through up-regulating the expressions of CDKN1A and CDKN1B mediated by activation of the FRα/c-SRC/ERK1/2/NFκB/TP53 signaling pathway. Although we have not completely finished the delineation of the signaling pathways involved in regulating FA-induced growth inhibitions in HT-29 and LoVo colon cancer cell lines, our preliminary data showed that FRα is the only isoform detected in HT-29 and LoVo and c-SRC as well as ERK were activated and the levels of TP53, CDKN1A and CDKN1B protein were increased in the FA-treated HT-29 and LoVo. These findings suggest that FA inhibited the growth of these colon cancer cells through a similar signaling pathway. The findings from the present *in vitro* and *in vivo* studies strongly suggest the potential applications of FA in the treatment of colon cancer.

## Methods

### Chemicals

FA, anti-vonWillebrand factor (VWF) antibody and anti-CDKN1B antibody were purchased from Sigma-Aldrich (St.Louis, MO). Trypsin-EDTA and kanamycin were purchased from Life Technologies (Grand Island, NY). Antibodies specific for G3PDH was purchased from GeneTex, Inc. (Hsinchu, Taiwan). Anti-TP53, p-ERK, ERK, IκBα, NFκB, and FR antibodies were purchased from Santa Cruz Biotechnology (Santa Cruz, CA). Antibodies specific for CDKN1A, p-IκBα and p-NFκB was purchased from Cell Signaling Technology, Inc. (Beverly, MA). c-SRC and p-c-SRC antibodies were purchased from Abcam (Cambridge, UK). PP2 was purchased from A. G. Scientific, Inc. (San Diego, CA). U0126 and caffeic acid phenethyl ester (CAPE) were purchased from Cayman Chemical (Ann Arbor, MI). MDM2 was purchased from Millipore (Temecula, CA).

### Cell culture and Cell transfection

COLO-205, HT-29 and LoVo purchased from ATCC were used in this study. These cells have performed STR-PCR profile at MB Mission Biotech. The cell was grown in RPMI-1640 supplemented with 10% fetal bovine serum (FBS) and kanamycine (100 ng/mL) in a humidified incubator (37 °C, 5% CO2). For transient transfection of the indicated constructs into COLO-205, jetPEI-HUVEC transfection reagent (Polyplus Transfection, Bioparc, France) was used and the transfection was performed according to the manufacturer’s protocol. Briefly, jetPEI-COLO-205/DNA mixture was added drop-wise into the DMEM+GlutamaxTM I medium (GIBCO) containing 2% FBS, mixed gently, and incubated in a humidified 37 °C incubator for 4 h. The growth medium was then replaced and the cell was incubated for the next 24 h.

### [3H]thymidine incorporation

The [3H]thymidine (Amersham Biosciences, UK) incorporation was performed as previously described^33^. Briefly, COLO-205 were applied to 24-well plates in growth medium (RPMI-1640 supplemented with 10% FBS, 100 ng/mL kanamycine). After COLO-205 had grown to 60–70% confluence, they were rendered quiescent by incubation for 24 h in RPMI-1640 containing 0.04% FBS. RPMI-1640 supplemented with 10% FBS and PBS with or without FA (0.1–10 μM) was added to the cell and the culture was allowed to incubate for 24 h. During the last 3 h of the incubation, [3H]thymidine was added at 1 μCi mL^−1^ (1 μCi = 37 kBq). Incorporated [3H]thymidine was extracted in 0.2 N NaOH and measured in a liquid scintillation counter.

### Flow cytometric analysis

The cell was transfected with pcDNA 3.1(+) or dominant-negative *TP53* (DN-*TP53*) construct (2.5 μg) for 5 h, rendered quiescent with serum free medium for 24 h, and then challenged with RPMI-1640 medium containing 10% FBS and PBS (control) or FA (10 μM). Cells were harvested at various time points after released with trypsin-EDTA, washed with PBS, and fixed in 70% ethanol at 4 °C for 1 h. Ethanol was removed and cell pellet was washed with PBS twice before staining. DNA content of nuclei was determined by staining nuclear DNA with a solution containing propidium iodine (2 mg/mL) and DNase-free RNase A (10 mg/mL) and measured using a fluorescence-activated cell sorter.

### Dominant-negative *TP53* construct

To amplification of the mutant (V143A) of *TP53*^18^,^34^, recombinant PCR was performed. Synthesized cDNA (SuperScript III First-Strand Synthesis System for RT-PCR; Invitrogen, Carlsbad, CA) from HUVEC was used as a template and Phusion high-fidelity DNA polymerase (Finnzymes Diagnostics, Finland) with 3’ to 5’ exonuclease activity was used in subsequent PCR. Forward primer 5’-ATGGAGGAGCCGAGTCAG and reverse 5’– TCAGTCTGAGTCAGGCCCTTC primers were used for amplified full-length wild type *TP53* (WT-*TP53*); then used two primer sets 1 and 2 (Table 1) were used to synthesize fragment A and fragment B. For each fragment, the amplified PCR product was purified and used as a mixed template for synthesizing *TP53*^V143A^ in the PCR reaction. The synthesized PCR product of *TP53*^V143A^ was purified, digested with restriction enzyme HindIII and EcoRV, and cloned into pcDNA 3.1(+) (Invitrogen). The *TP53*^V143A^ sequence fidelity was confirmed by ABI PRISM 377 DNA Analysis System.

### MTT assay

Cell growth were estimated by a modified MTT [3-(4,5-Dimethyl-2- thiazolyl)-2,5-diphenyl-tetrazolium bromide] (USB/Amersham Life Science) assay after the cell was treated with FA (10 μM) for 24 h (cell viability) or daily with FA (10 μM) for six days (cell growth).

### Trypan blue assay

As a measurement of cell viability, the cell was seeded onto 12 well dish and grown in RPMI-1640 medium containing 10% FBS. Media without (control) or with FA (10 μM) were changed daily until cell counting and were released with trypsin–EDTA (GIBCO). The cell suspension was mixed with equal volume of 0.4% trypan blue solution (Sigma-Aldrich) and incubated at room temperature for 3 min. Cells were counted using the dual-chamber hemocytometer under microscope. Viable or nonviable cells were recorded from three independent counts for analysis.

### Protein extraction and Western blot analysis

The cell was seeded onto 6 cm dishes and grown in RPMI-1640 supplemented with 10% FBS. After cells had grown to subconfluence, they were rendered quiescent by incubation for 24 h in RPMI-1640 medium containing 0.04% FBS. The cell was released from quiescence with culture medium containing 10% FBS, treated with FA at indicated concentrations or PBS for the control group, washed with PBS, and then lysed in lysis buffer (0.5 M Tris-HCl, pH 6.8, and 0.4% sodium dodecyl sulfate). Western blot analyses were applied to determine the protein levels in cells; the electrophoresis was performed using 8% or 12% sodium dodecyl sulfate-polyacrylamide gel (3 h, 70 V). Separated proteins were transferred onto polyvinyl difluoride membranes (4 h, 400 mA), treated with 5% skim milk (Anchor, Auckland, NZ) to block the nonspecific IgGs and incubated overnight at 4 °C with specific antibody. The blot was then incubated with anti-mouse or anti-rabbit IgG (Jackson ImmunoResearch Laboratories) conjugated to horseradish peroxidase for 1 h. Subsequently, the polyvinyl difluoride membrane was developed with chemiluminescence reagent (PerkinElmer Life Sciences, Boston, MA). The intensity of each band was quantified by densitometry analysis using Image Pro Plus 4.5 software. In each figure, the gels have been run under the same experimental conditions and cropped blots were shown. The methods were carried out in “accordance” with the approved guidelines.

### Reverse Transcription-PCR (RT-PCR)

Total cellular RNA was extracted from cells with Trizol (Life Technologies, Carlsbad, CA) according to the manufacturer’s protocol^22^. The RNA pellet was washed with 75% cold ethanol, air dried, and re-dissolved in 20 μL diethyl pyrocarbonate-treated water. Two μg of total RNA were used in a total of 20 μL reaction volume as a template for PCR amplification. PCR was done under standard conditions in 20 μL of 10 mM Tris (pH 8.3), 40 mM KCl, 1.5 mM MgCl_2_, 250 μmol/L dNTP, 10 μM of each primer (sense and antisense), and 1 unit Taq DNA polymerase. The PCR primer sequences for FRα, FRβ, FRγ, and FRδ were listed in Table 1. The PCR products were electrophoresed on a 1.5% agarose in 0.5X Tris-acetate/EDTA buffer and stained with ethidium bromide solution (Roche, Mannheim, Germany).

### Antisense oligonucleotide

The antisense oligonucleotide (AS) sequence of FRα: 5’-TGTTGTCATCCGCTGAGCCAT-3’ was designed by previous study^35^, which is complementary to the region of the sense template of FRα. The scramble oligonucleotide (Sc) sequence of FRα: 5’-GTCTCGTTCGTATACGCTACG-3’ was used for the control. Briefly, cells were plated per well in 6 cm plate and washed twice with serum-free MEM. AS or Sc was added in RPMI-1640 containing 5% FBS without antibiotic, and the final concentration of oligonucleotide was 10 μM each. Cells were incubated with AS or Sc for 4 h. Transfections were terminated by the addition of RPMI-1640 containing 10% FBS.

### Chromatin immunoprecipitation (ChIP) assay

The ChIP assay was performed as previously described^19^. Briefly, COLO-205 was fixed with 1% formaldehyde in room temperature for 10 min, and then stopped by glycine (final concentration of 0.125 M). The cell was washed twice with ice-cold PBS, resuspended, and then homogenized in 0.5 mL swelling buffer (5 mM 1,4-piperazine diethane sulfonic acid, pH 8.0; 0.5% Triton X-100; 0.5 mM protease inhibitor (G-Biosciences, LA, USA)). The nuclei pellet was sonicated in 0.5 mL sonication buffer (10 mM EDTA; 50 mM Tris-HCl, pH 8.0; 0.5 mM PMSF) plus protease inhibitor (G-Biosciences). A solution containing 0.1% SDS and 85 mM KCl was added to reduce the nonspecific binding. Samples were divided into two equal amounts and individually incubated for 1 h at 4 °C with 1.5 μg of anti-NFκB antibody (Santa Cruz Biotechnology) or 1.5 μg of normal mouse IgG (Santa Cruz Biotechnology) as a negative control, added with 30 uL of protein A agarose/salmon sperm DNA slurry (Upstate, Temecula, CA), and then incubated at 4 °C overnight with constant rotation. The protein A agarose/antibody/chromatin complex was washed and then dissociated using the Upstate ChIP protocol. DNA was purified using the Geneaid PCR purification kit (Geneaid, Taipei, Taiwan) and eluted in a 50 μL elution buffer (10 mM Tris-HCl pH 8.5). Two μL of the DNA was assayed in a PCR cycle. The NFκB binding site within the *TP53* promoter was analyzed in MOTIF Search (http://motif.genome.jp/) and the primers designed using Primer-BLAST of National Center for Biotechnology Information (Bethesda, MD) were listed in Table 1. All the PCR products were analyzed using 2% agarose gels containing ethidium bromide (New Taipei city, Taiwan).

### Nuclear extraction

To examine the effect of FA on nuclear translocation of NFκB, the NE-PER® nuclear and cytoplasmic extraction reagents (Thermo Fisher Scientific, Rockford, IL) were used and the extraction was performed according to the manufacturer’s protocol. Briefly, the cell pellet was disrupted in cytoplasmic extraction reagent I buffer plus protease inhibitor (G-Biosciences), and then lysed in cytoplasmic extraction reagent II buffer to release cytoplasmic proteins. After being centrifuged at 13,000 rpm for 10 min at 4 °C, the insoluble pellet, which contained nuclear proteins, was washed with ice-cold PBS twice, and then incubated in nuclear extraction reagent buffer plus protease inhibitor (G-Biosciences).

### Tumor growth assay

The tumor growth assay was performed as previously described^36^. The COLO-205 cells were implanted by injection of a tumor suspension (3 × 10^6^ cells in 0.1 mL RPMI 1640) subcutaneously in the flanks of male severe combined immunodeficiency (SCID) mice (BALB/c-nu, National Animal Center, Taiwan). Each mouse was implanted with one bolus of tumor cells on day 7. Dosing (i.p.) with FA was begun 7 days after implantation (day 0), when the tumors reach ~7  mm^3^ in volume. Either the vehicle or FA at a dose of 100 μg or 300 μg was given i.p. daily. All *in vivo* procedures were approved by the Taipei Medical University Animal Care and Use Committee.

### Statistical analysis

Values represent the means ± s.e.mean. Three to four samples were analyzed in each experiment. Comparisons were subjected to t-test and one way analysis of variance (ANOVA) followed by Fisher’s least significant difference test. Significance was accepted at *P* < 0.05

## Additional Information

**How to cite this article**: Kuo, C.-T. *et al*. Folic acid inhibits COLO-205 colon cancer cell proliferation through activating the FRα/c-SRC/ERK1/2/NFκB/TP53 pathway: *in vitro* and *in vivo* studies. *Sci. Rep*. **5**, 11187; doi: 10.1038/srep11187 (2015).

## Supplementary Material

Supplementary Information

## Figures and Tables

**Table 1:** Primer sequences and amplicon size used in this study.

**Name of Genes**		**Sequence**	**Amplicon (bp)**
FRα	F’	AGCCCATAAGGATGTTTCCTA	356
	R’	TTTCATTGCACAGAACAGTG	
FRβ	F’	CACCTCCCGCCTGTACAACTT	489
	R’	ATCTCACCAGCATTCACATGC	
FRγ	F’	ATGGACATGGCCTGGCAGATG	531
	R’	GGTGCTGCAGAGGGCCCCGGCCGG	
FRδ	F’	CCTCACGACAAGCTGGGAAG	376
	R’	AGTCTTCTCACACAGGTCAGC	
p65 binding site within p53 promoter	F’	CCTCACGACAAGCTGGGAAG	151
	R’	CCTCACGACAAGCTGGGAAG	
WT-p53	F’	ATGGAGGAGCCGAGTCAG	1184
	R’	TCAGTCTGAGTCAGGCCCTTC	
Dominant negative p53 fragment A	F’	CTTAAGCTTGCCGCCATGGAGGAG	420
	R’	CACAGATGCGCAGGGCAGGT	
Dominant negative p53 fragment B	F’	ACCTGCCCTGCGCAGCTGTG	764
	R’	GTGCTGGATATCTCAGTCTGAGTC	
