# Supplementary material for: Folic acid inhibits COLO-205 colon cancer cell proliferation through activating the FRα/c-SRC/ERK1/2/NFκB/TP53 pathway: in vitro and in vivo studies
Source: Sci Rep. 2015 Jun 9;5:11187. doi: 10.1038/srep11187 (PMC4460902; doi:10.1038/srep11187)
Supplement: Supplementary Information [file srep11187-s1.pdf]

Folic acid inhibits COLO-205 colon cancer cell proliferation through activating the FRα/c-SRC/ERK1/2/NFκB/TP53 pathway: *in vitro* and *in vivo* studies

Chun-Ting Kuo[1], Chieh Chang[2] and Wen-Sen Lee[1,3,4*]

[1]Graduate Institutes of Medical Sciences, College of Medicine, Taipei Medical University, Taipei 110, Taiwan
[2]School of Pharmacy, Taipei Medical University, Taipei 110, Taiwan
[3]Department of Physiology, School of Medicine, College of Medicine, Taipei Medical University, Taipei 110, Taiwan
[4]Cancer Research Center, Taipei Medical University Hospital, Taipei 110, Taiwan

***Corresponding author at:** Graduate Institute of Medical Sciences, Taipei Medical University, 250 Wu-Hsing Street, Taipei 110, Taiwan. Phone: +886-2-27361661 ext. 3221. Fax: +886-2-23778620. E-mail: wslee@tmu.edu.tw

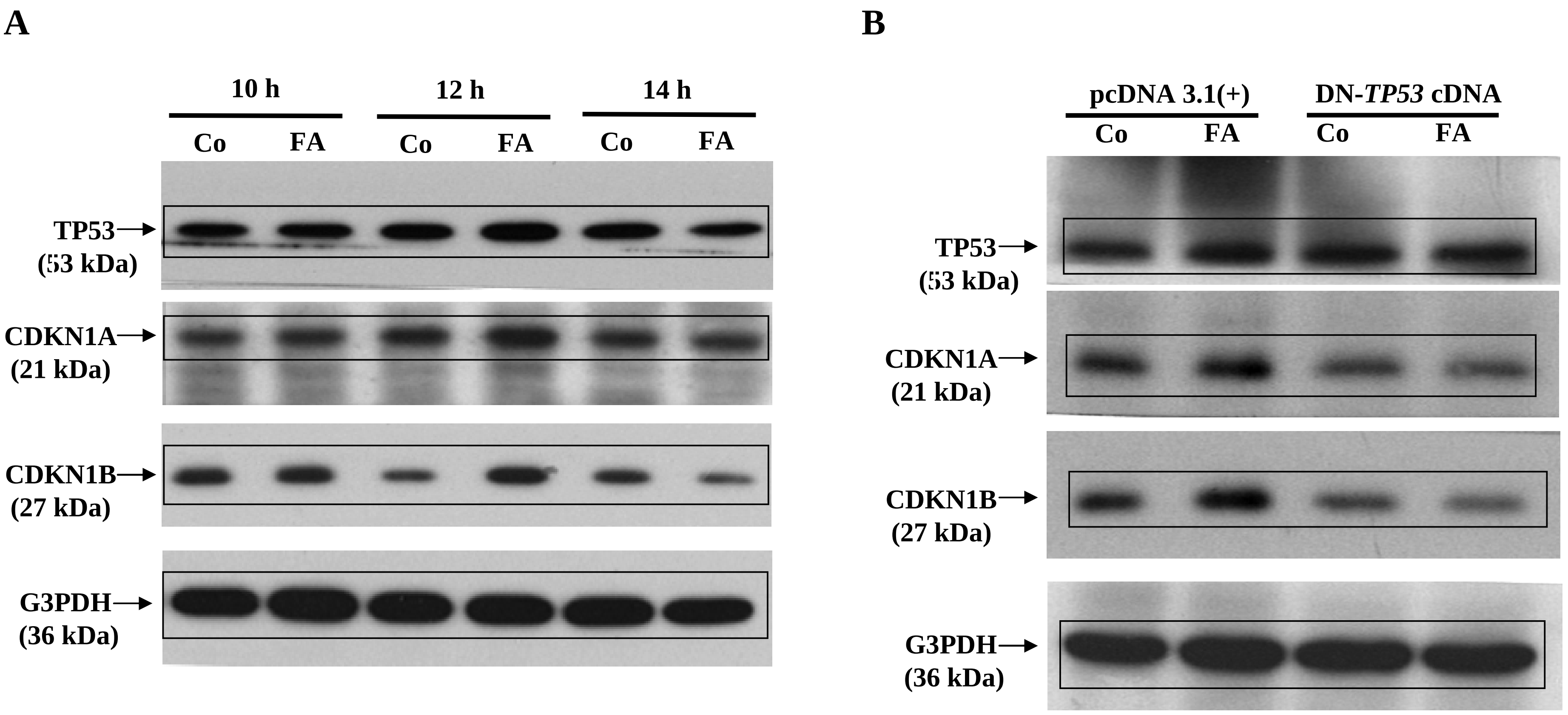

Suppl. Fig. 1. The entire gel pictures of Figures 1 and 2 in the text. (A) Corresponding to Fig. 1C. (B) Corresponding to Fig. 2A. The cropping lines and the molecular weight of each protein were indicated .

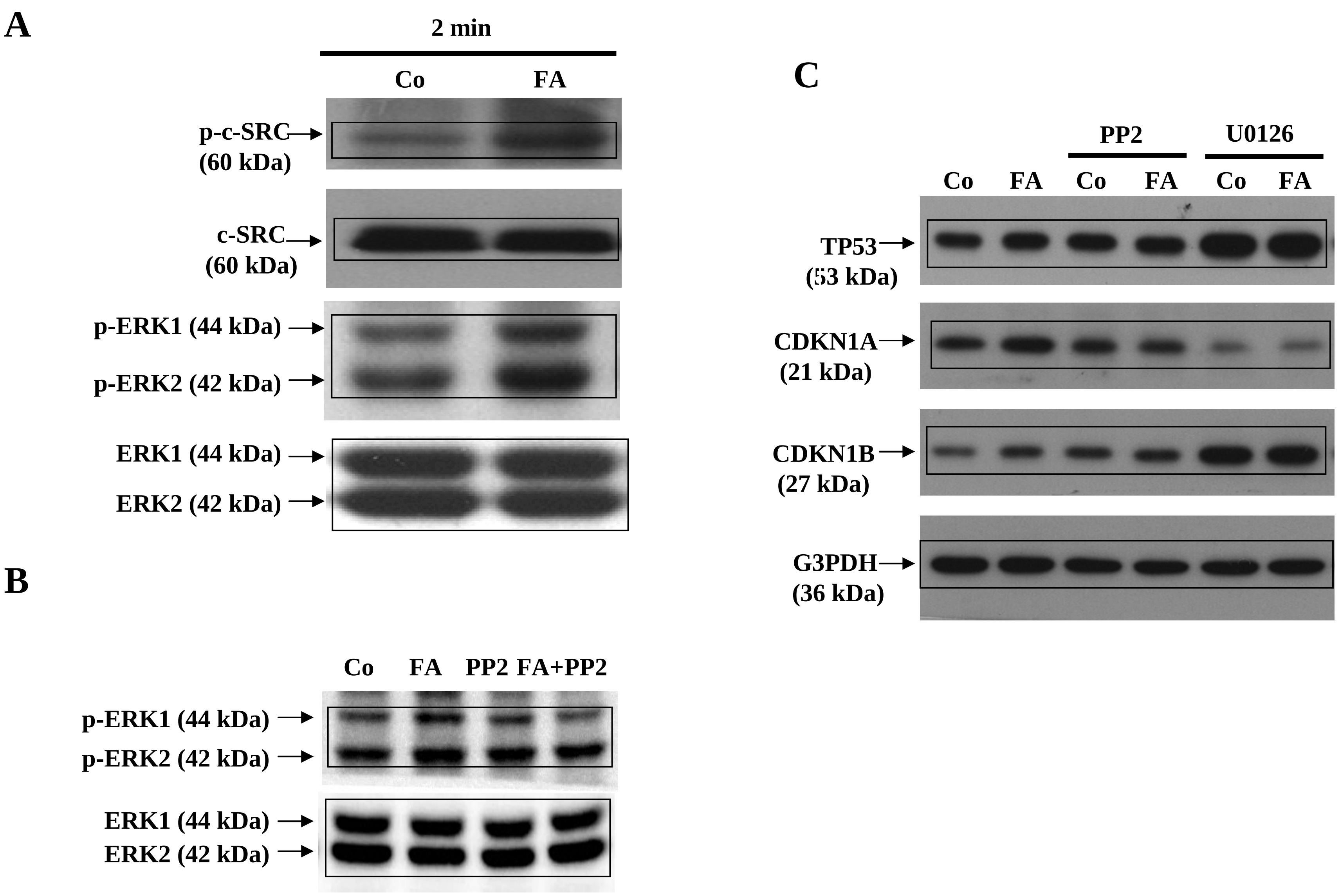

Suppl. Fig. 2. The entire gel pictures of Figure 3 in the text. (A) Corresponding to Fig. 3A. (b) Corresponding to Fig. 3B. (c) Corresponding to Fig. 3C. The cropping lines and the molecular weight of each protein were indicated .

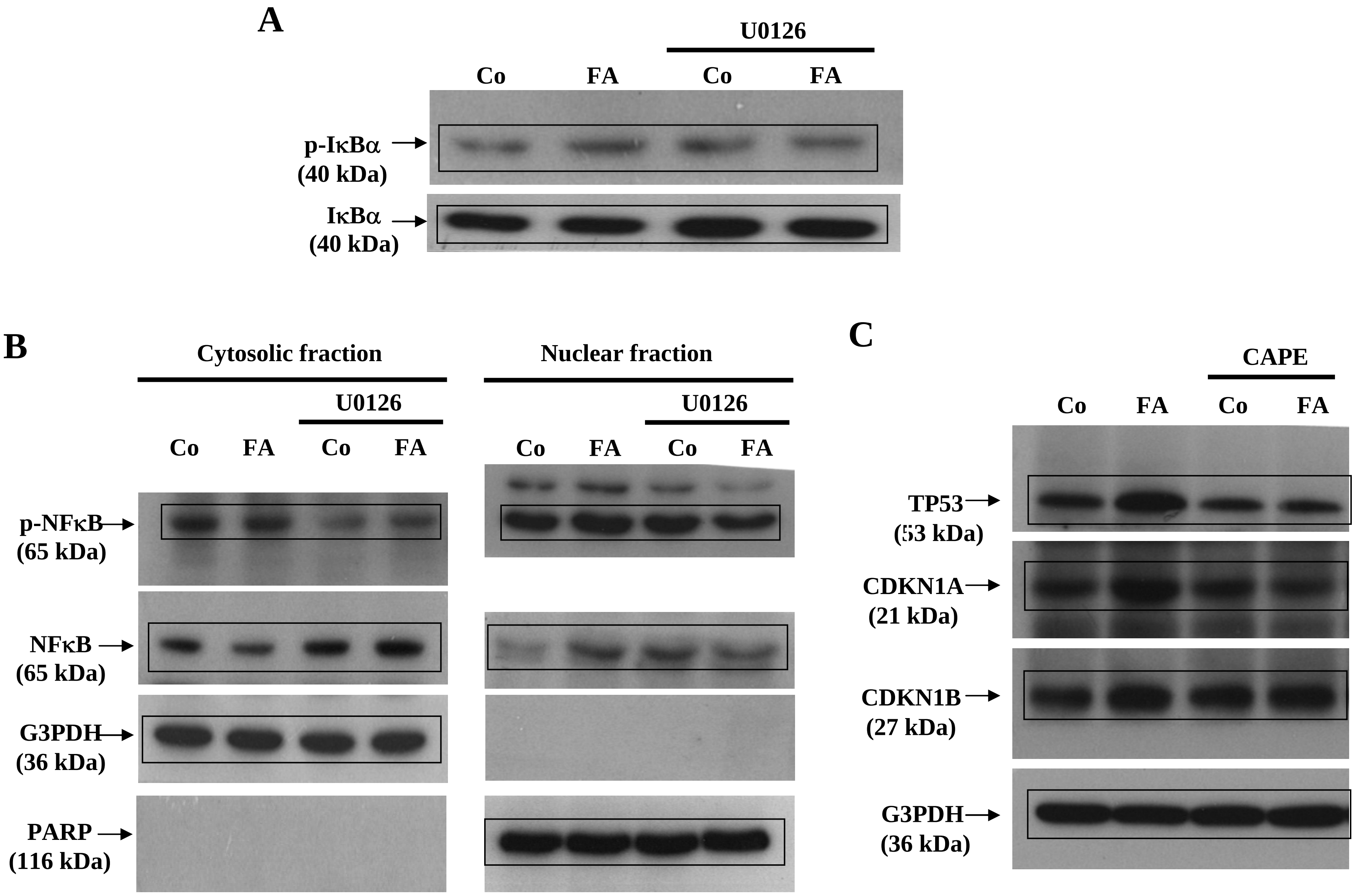

Suppl. Fig. 3. The entire gel pictures of Figure 4 in the text. (A) Corresponding to Fig. 4A. (B) Corresponding to Fig. 4B. (C) Corresponding to Fig. 4C. The cropping lines and the molecular weight of each protein were indicated .

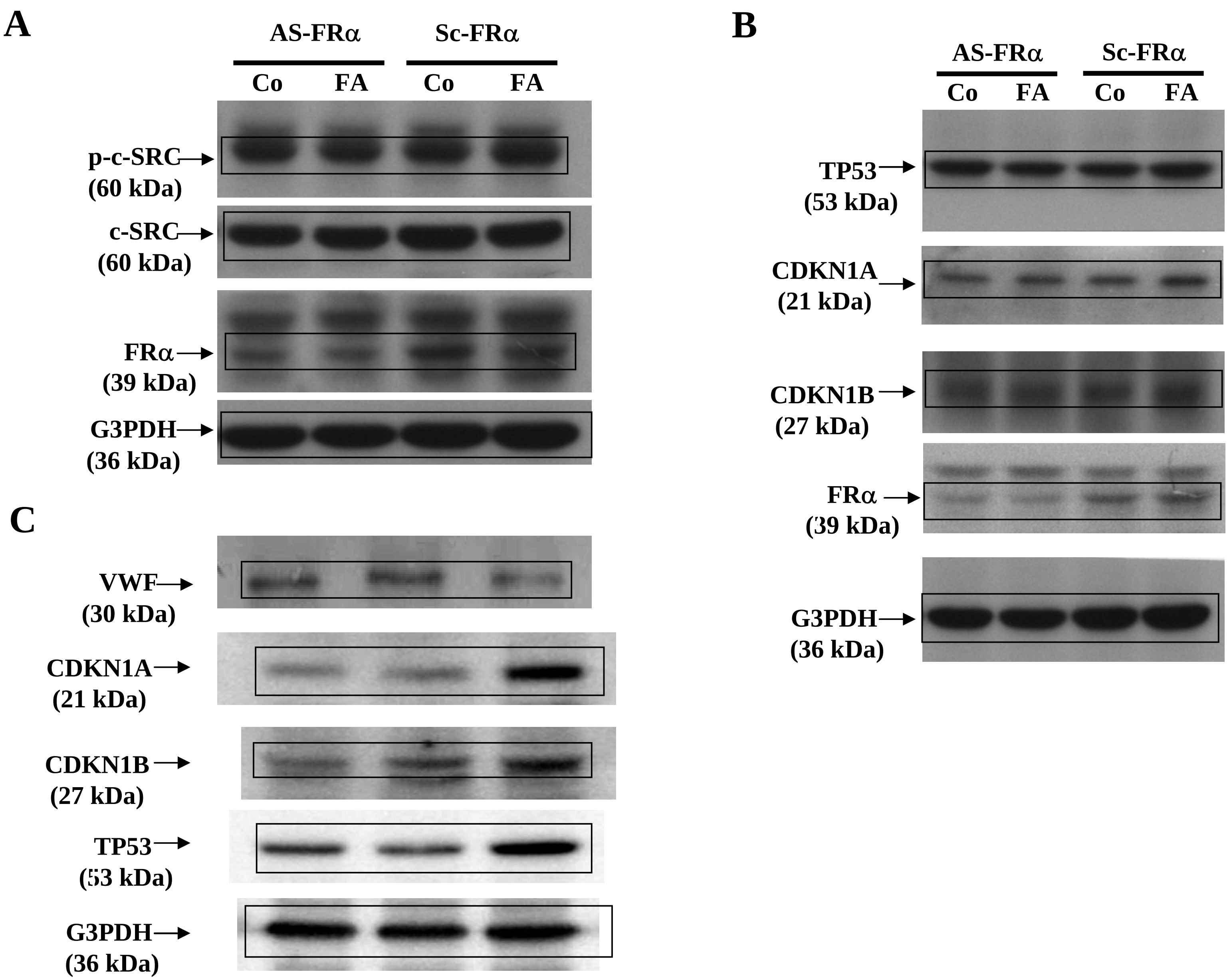

Suppl. Fig. 4. The entire gel pictures of Figures 5 and 6 in the text. (A) Corresponding to Fig. 5B. (B) Corresponding to Fig. 5C. (C) Corresponding to Fig. 6A. The cropping lines and the molecular weight of each protein were indicated.

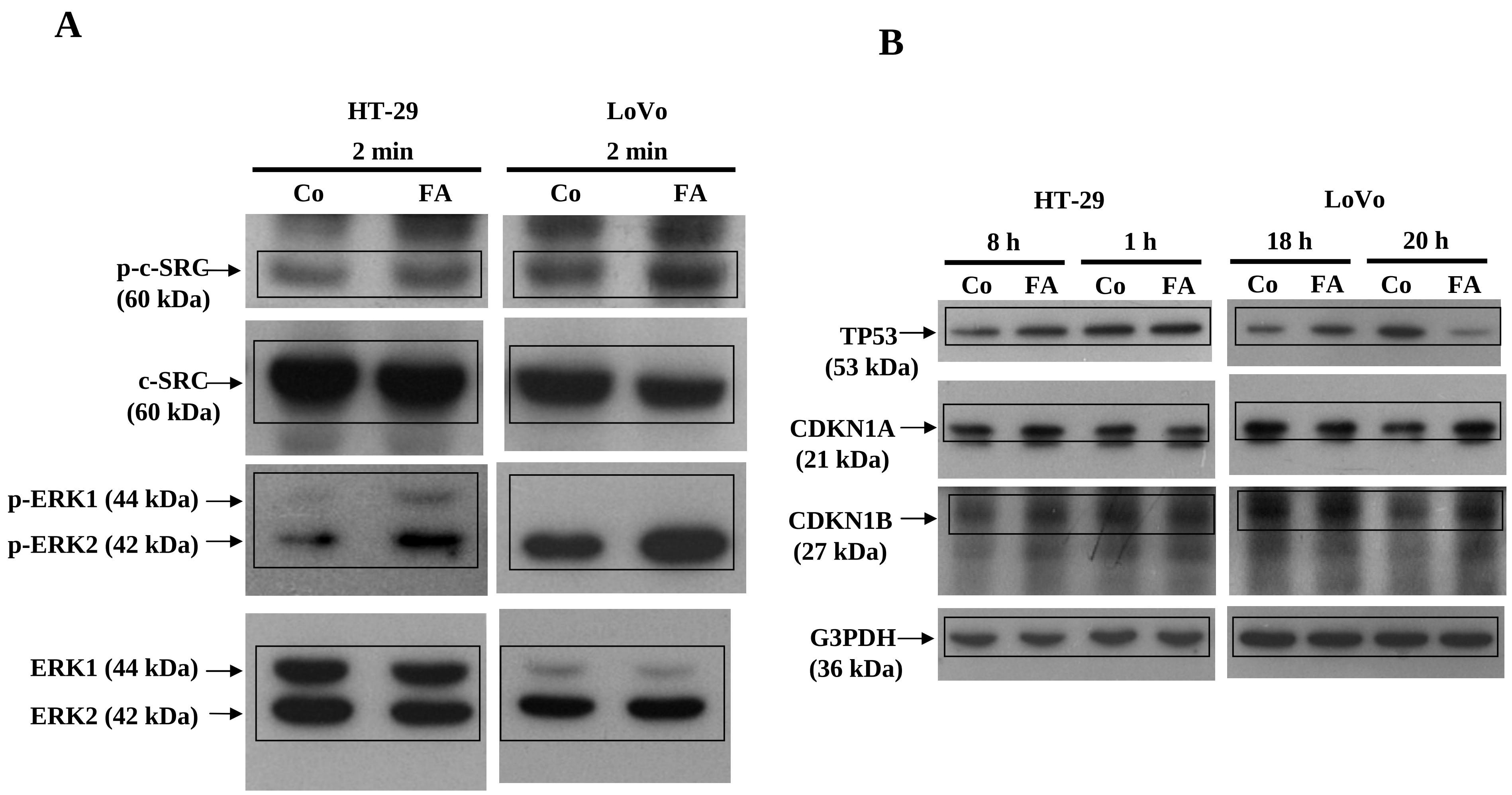

Suppl. Fig. 5. The entire gel pictures of Figure 7 in the text. (A) Corresponding to Fig. 7C. (B) Corresponding to Fig. 7D. The cropping lines and the molecular weight of each protein were indicated .

A

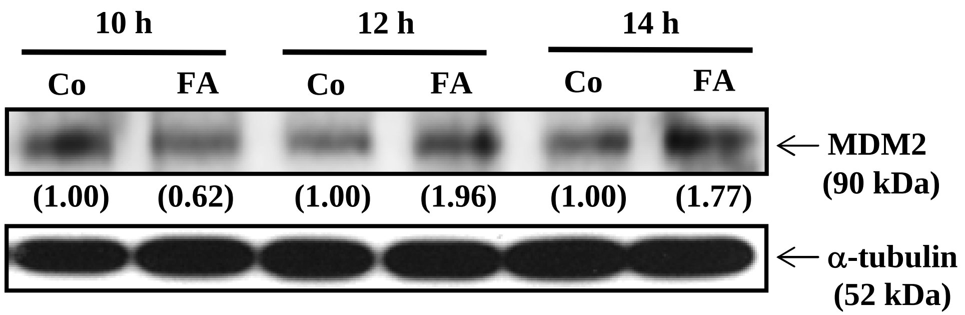

B

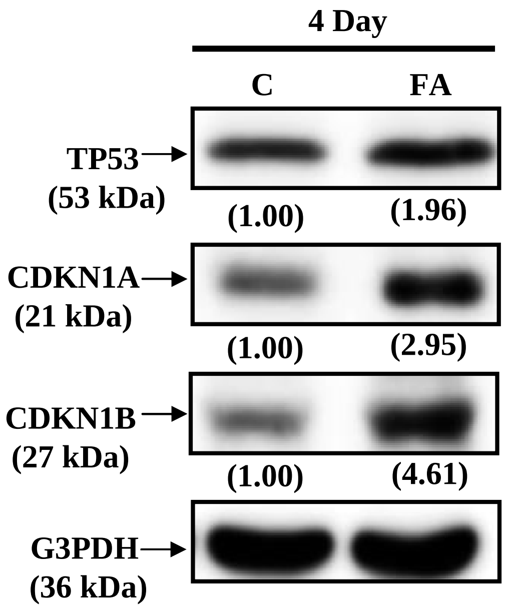

Suppl. Fig. 6. Effects of FA treatment on the levels of MDM2, TP53, CDKN1A and CDKN1B protein in COLO-205. (A) Treatment with FA (10 μM) for 12 or 14 h increased the level of MDM2 protein in COLO-205. (B) The increased levels of TP53, CDKN1A and CDKN1B protein were observed in the COLO-205 after four days continuous treatment with FA (10 μM).
